# Transmission of societal stereotypes to individual-level prejudice through instrumental learning

**DOI:** 10.1073/pnas.2414518121

**Published:** 2024-11-01

**Authors:** David T. Schultner, Benjamin S. Stillerman, Björn R. Lindström, Leor M. Hackel, Damaris R. Hagen, Nils B. Jostmann, David M. Amodio

**Affiliations:** ^a^Department of Psychology, University of Amsterdam, Amsterdam 1001NK, The Netherlands; ^b^Department of Clinical Neuroscience, Division of Psychology, Karolinska Institutet, Stockholm 17177, Sweden; ^c^Department of Psychology, New York University, New York, NY 10012; ^d^Department of Psychology, University of Southern California, Los Angeles, CA 90089

**Keywords:** stereotypes, prejudice, learning, instrumental, computational

## Abstract

How are societal stereotypes transmitted to individual-level group preferences? We propose that exposure to a stereotype, regardless of whether one agrees with it, can shape how one experiences and learns from interactions with members of the stereotyped group, such that it induces individual-level prejudice—a process involving the interplay of semantic knowledge and instrumental learning. In a series of experiments, participants interacted with players from two groups, described with either positive or negative stereotypes, in a reinforcement learning (RL) task presented as a money-sharing game. Although players’ actual sharing rates were equated between groups, participants formed more positive reward associations with players from positively stereotyped than negatively stereotyped groups. This effect persisted even when stereotypes were described as unreliable and participants were instructed to ignore them. Computational modeling revealed that this preference was due to stereotype effects on priors regarding group members’ behavior as well as the learning rates through which reward associations were updated in response to player feedback. We then show that these stereotype-induced preferences, once formed, spread unwittingly to others who observe these interactions, illustrating a pathway through which stereotypes may be transmitted and propagated between society and individuals. By identifying a mechanism through which stereotype knowledge can bypass explicit beliefs to induce prejudice, via the interplay of semantic and instrumental learning processes, these findings illuminate the impact of stereotype messages on the formation and propagation of individual-level prejudice.

## Significance

How do social stereotypes that exist in society transform into individual-level prejudices? In a series of experiments, we show that stereotype exposure shapes how we learn about group members in direct social interactions and that this learning bias predicts the formation of group preferences. We further show that, once learned, these group preferences are transmitted to naïve observers who merely witness interactions between stereotyped group members and a person with stereotype knowledge. Finally, we show that this pattern of prejudice formation and propagation occurs even when people view the stereotype as unreliable and attempt to inhibit its influence. Together, these studies reveal a mechanism through which stereotypes may be transmitted and propagated between society and individuals.

How do explicit stereotypic messages about social groups become internalized in an individual’s own preferences and behaviors? When a politician refers to a group as “criminals and rapists,” as Donald Trump famously did during his 2015 campaign announcement, people may dismiss the epithets as mere rhetoric. Yet such messages may nevertheless be encoded in the listener’s memory. We asked whether such knowledge, even when dismissed, can shape how people subsequently perceive and learn from members of the targeted group in direct interactions, such that it transforms into personal group preferences—a process representing the transmission of prejudice from societal-level stereotypes to individual-level attitudes.

To understand how stereotype knowledge may transform into individual-level prejudice through social interaction, we considered the interplay of learning mechanisms underlying stereotype knowledge and social-interactive impression formation (1–3). Stereotypes are societally held beliefs about a group and its members, encoded in semantic memory (4–6). By providing expectancies for group members’ behaviors, stereotypes can shape how we perceive and interpret a person’s actions (7–10). However, like other forms of semantic knowledge, mere knowledge of a stereotype does not imply its endorsement: most low-prejudice individuals explicitly reject social stereotypes and inhibit stereotype effects on their judgments and behaviors (8, 11–13). This longstanding view within intergroup bias research suggests that an individual’s personal beliefs are insulated from their knowledge of societal stereotypes (10, 14). From this perspective, exposure to a stereotype message should not, by itself, induce individual-level prejudice.

Here, however, we considered an unexplored possibility: If stereotypes provide expectancies for a group member’s behavior, can stereotype knowledge inadvertently bias how we experience and learn about group members during direct social interactions? In direct interactions, a perceiver learns about a group member through the exchange of action and feedback—a process characterized by instrumental learning (i.e., reward reinforcement) (1, 3, 15). In contrast to stereotype knowledge, represented by semantic concepts, instrumental learning forms incrementally through repeated interaction and feedback, encoded in terms of reward value, and is expressed in choice behaviors that reflect an individual’s personal, internalized preferences (16–18). Furthermore, whereas stereotype knowledge is explicit and easily inhibited in overt responses, instrumental learning is considered nondeclarative, such that it can form without explicit awareness of learning contingencies (19, 20). As a result, it may be especially difficult for a learner to detect or inhibit unwanted influences on the impressions they form of people through instrumental learning in direct interactions.

How might stereotypes influence instrumental learning? Instrumental learning can be shaped by priors, such as past experiences or knowledge, which can affect one’s expectations about feedback and the degree to which a reward association is updated (17, 21). If stereotypes function as priors in instrumental learning, then exposure to a stereotype message may also bias reward expectancies associated with a group and the degree to which this reward association is updated in response to a group member’s feedback. This process, involving the interplay of semantic and instrumental learning, would represent a pathway through which stereotype knowledge may bypass explicit egalitarian beliefs to produce individual-level prejudice.

Based on this analysis, we hypothesized that stereotype messages can induce personal group-based preferences through two concerted processes: First, exposure to a positive or negative stereotype sets initial expectations (i.e., *priors*) for a group member’s behavior; second, stereotypes influence learning—that is, the degree to which reward representations are updated in response to feedback across repeated interactions (i.e., the *learning rate*)—such that updating occurs differently for members of positively and negatively stereotyped groups.

We tested this *stereotype learning* hypothesis across eight experiments in which we predicted that stereotype descriptions of groups would influence participants’ instrumental learning during direct interactions with group members, even when participants explicitly dismiss the stereotype. We examined this effect in participants’ behaviors and tested our hypothesis using computational modeling, and then further examined how such biases, once acquired and expressed, may spread to others who observe these direct interactions.

**Fig. 1.**
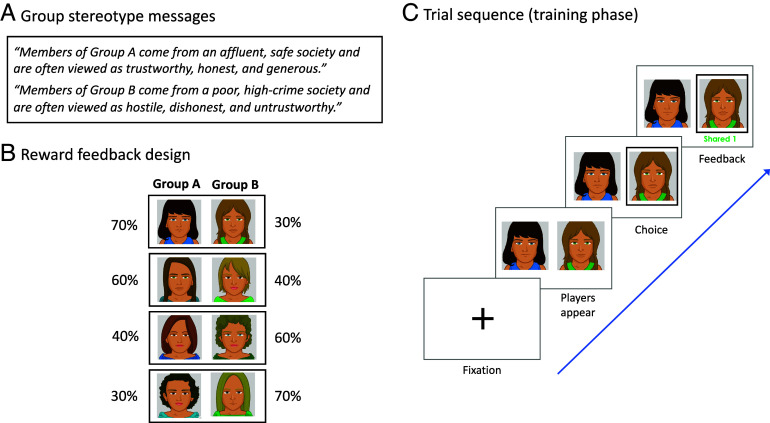
Schematic of the sharing game training phase. (*A*) Participants were exposed to positive and negative stereotype messages regarding each group and then (*B*) interacted with members of two groups who shared points at fixed reward rates (70%, 60%, 40%, or 30%). Group labels (A and B), member features (e.g., hair, shirt color), and gender were counterbalanced across participants. (*C*) On each trial, participants chose between players (group members) and received reward feedback.

In experiments 1 to 3, participants interacted with people from two different social groups in an online point-sharing game. These groups were labeled “Group A” and “Group B” (counterbalanced) in the task, ostensibly to maintain their anonymity, but described using positive or negative societal stereotypes associated with White and Black Americans, respectively (14). Group A was characterized as coming from a relatively wealthy, safe, and highly educated community, whereas Group B’s community was characterized as relatively poor and uneducated and with a high crime rate (Fig. 1*A*; *SI Appendix*). This approach allowed us to isolate effects of stereotypes on learning while controlling for participants’ existing group knowledge. Despite these group descriptions, participants were told that individual group members varied in their tendency to share points during the game and therefore, given participants’ explicit goal to earn points, they should attend to the individual sharing rate of each player. Participants then completed a point-sharing game with members of both groups, receiving cash payouts for their winnings.

The sharing game was adapted from a widely used probabilistic reward reinforcement learning (RL) task (22). In this version, participants interacted with four players from each group. Within groups, each player shared points at a different fixed rate (70%, 60%, 40%, or 30%), but average sharing rates were equated between groups (Fig. 1*B*). Participants first completed a training phase, in which they could learn from feedback on each trial and, by choosing players who shared, earn points that would be converted to a cash bonus. On each round of training (160 trials), participants were presented with a preset pair of players—one from each group, with fixed complementary sharing rates (e.g., Players A and B)—and chose, via button press, with whom to interact (Fig. 1*C*). Reward feedback, displayed immediately beneath the image of the chosen player, indicated whether the chosen player shared (+1 or 0 points). Participants knew that only one player would share on each round.

Following the training phase, participants completed the test phase (96 trials), which provided a readout of their learning. In the test phase, participants viewed and selected between all possible pairs of Group A and B members. This allowed us to assess participants’ choice preferences between novel pairs of players at every combination of reward rate. Hence, the test phase provided a fine-grained behavioral assessment of learned reward associations with each member of the two groups (22). Although feedback was not provided to prevent further learning, participants were told they would receive cash payout for their test phase choices following task completion.

## Results

**Fig. 2.**
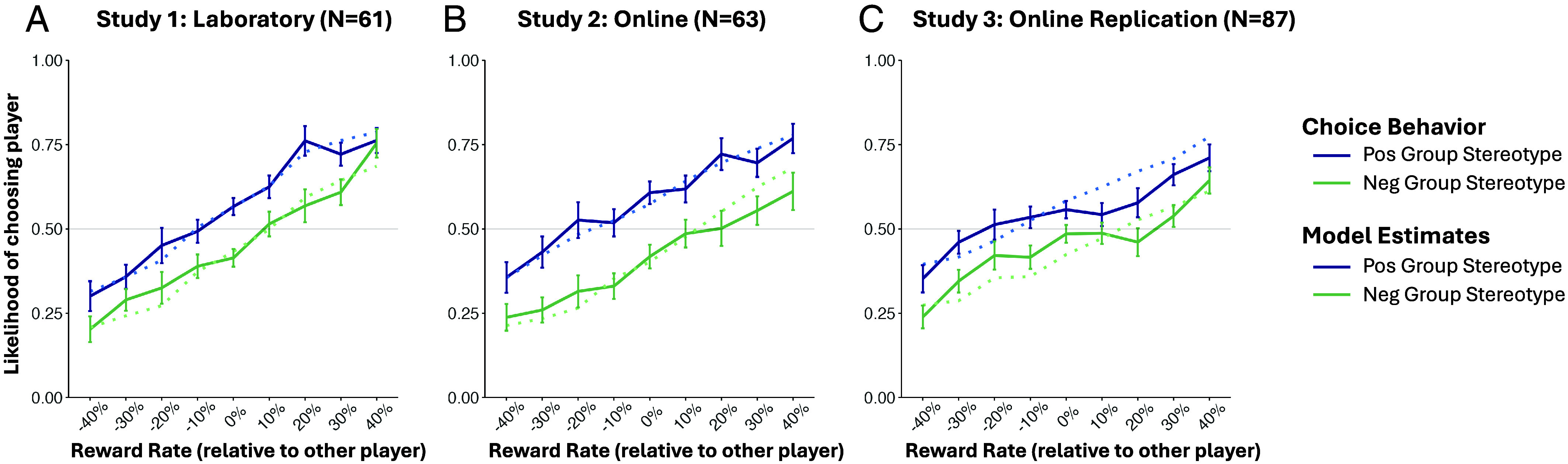
Behavioral choice preferences during the test phase in Studies 1–3 as a function of reward rate and group stereotype (Panels *A*–*C*). Participants’ choices (solid lines) demonstrated both successful learning of rewards and a group bias. Reward rate (x axis) represents the actual reward rate of a given player minus the actual reward rate of the alternative player in a trial. Error bars indicate SE. Dotted lines show estimates simulated from the stereotype-learning model, which combined group-based priors and separate learning rates.

In Study 1 (*N* = 61 laboratory participants), we tested whether stereotypic group descriptions influenced participants’ choices of individual players, despite equivalent sharing rates between groups—the hallmark of group-based prejudice. Analysis of test phase behavior showed that while participants learned the general pattern of rewards, choosing players with higher sharing rates on average (*B* = 2.68, *SE* = 0.19, Wald *z* = 14.43, *P* < .001; all tests two-tailed), their choices were also significantly affected by players’ group membership (*B* = 0.52, *SE* = 0.06, Wald *z* = 9.33, *P* < .001; Fig. 2*A*). This effect of group membership emerged despite participants’ extensive direct experience with players’ actual sharing rates, which were equated between groups and thus contradicted the stereotypes, as well as the monetary incentive to choose accurately. These results revealed that choice preferences were guided by the group stereotype as well as actual reward feedback.

**Fig. 3.**
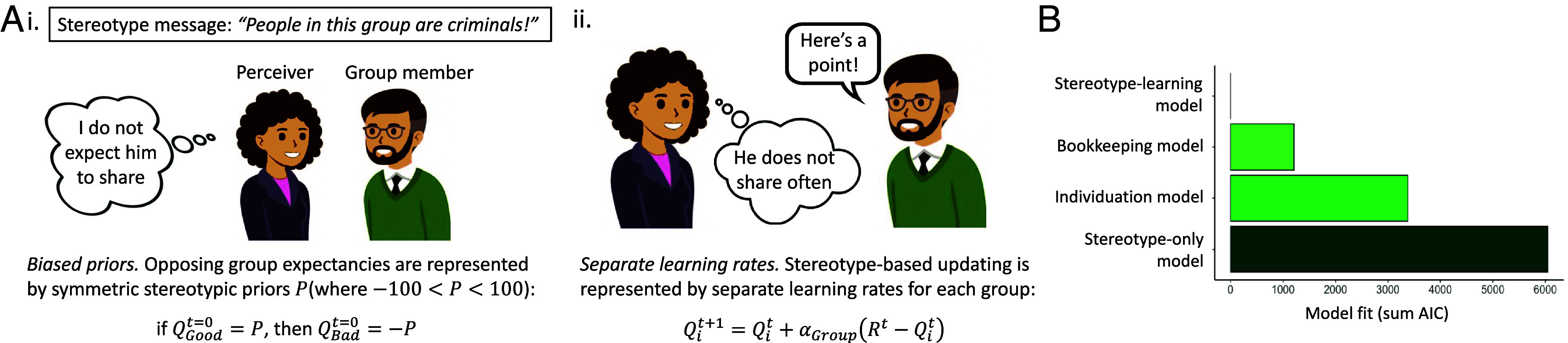
(*A*) According to the stereotype-learning model, i) a stereotype message creates a positive or negative expectancy (prior) for a group member’s behavior, and ii) in subsequent interactions, perceivers update the value of positively and negatively stereotyped group members with separate learning rates. (*B*) Model comparison (shown for Study 1) indicated the stereotype learning model fit best to data compared with other plausible models of stereotyping and impression formation.

Next, to test our specific hypothesis that this effect involved the influence of stereotype knowledge on instrumental learning, we fit behavior to a computational model specifying this process, adapted from (23). We conceptualized stereotype effects on group expectancy as separate *priors* for positively and negatively stereotyped groups, which set participants’ initial choice tendencies. Stereotype effects on learning (i.e., the updating of reward associations) were represented by separate *learning rates* for positively and negatively stereotyped groups. Thus, according to this hypothesized *stereotype learning model* (Fig. 3), the behavioral effects of stereotypes on instrumental learning reflect a combination of divergent group priors and separate group learning rates.

We compared the stereotype learning model with alternatives representing existing models of stereotyping and impression formation: a) a *bookkeeping model* (24, 25), in which new learning incrementally replaces the stereotype (biased priors and a single, unbiased learning rate), b) an *individuation model*, in which learning is based only on players’ actual behavior (a single learning rate and no priors), and c) a *classic stereotyping model*, in which stereotypes determine responses without learning (biased priors with no learning), in addition to other plausible RL and Bayesian accounts (see *Materials and Methods* and *SI Appendix* for model specifications and results). Model comparisons indicated that the stereotype learning model, which included stereotype priors and separate group learning rates, was most consistent with observed behavior, supporting our hypothesis (Fig. 3*B*; model fits in *SI Appendix*, Table S2).

This effect was replicated in two online experiments (Study 2: *N* = 62; Study 3: *N* = 87): In both, stereotypic group descriptions again significantly influenced participants’ test phase choice preferences (Study 2: *B* = 0.79, *SE* = 0.06, Wald *z* = 13.86, *P* < .001; Study 3: *B* = 0.48, *SE* = 0.05, Wald *z* = 9.58, *P* < .001), in addition to player’s actual reward rates (Fig. 2 *B* and *C*; *SI Appendix*). Again, this group bias emerged despite equivalent average reward rates between groups, participants’ explicit goal to individuate, and the financial incentive to choose players based on their actual behavior.

Computational modeling of Study 2 and 3 data each replicated the results of Study 1, such that choice behavior was most consistent with a model that included group-based priors and separate group learning rates (*SI Appendix*). Using combined data from Studies 1 to 3, parameter estimates of priors and group-specific learning rates, derived from the stereotype learning model, were submitted to a regression predicting group-based choice behaviors. Results indicated that the group bias in preferences reflected stereotype-based priors as well as insufficient updating for the negatively stereotyped group; that is, initial expectancies for the negatively stereotyped group were lower, relative to the positive group, and were not sufficiently updated in response to group members’ actual reward feedback (*SI Appendix*).

Study 3 was designed to address three additional aims. The first was to establish that stereotype descriptions were encoded in semantic memory. Participants completed a task in which they sorted stereotype traits used in the group descriptions to corresponding group labels. Classification accuracy for group stereotypes was significantly greater than chance (*M* = 75.02%; *t* = 7.87, 95% CI[0.68;0.78], *df* = 74, *P* < .001), indicating that stereotype descriptions were indeed encoded in memory.

The second aim was to test whether participants were aware of the stereotype effect on their choice preferences. To this end, we assessed participants’ subjective estimates of player sharing rates following completion of the sharing game. The subjective estimates were significantly predicted by the group stereotype, *B* = 31.31, *SE* = 8.49, *t* = 3.69, *P* < .001, independently of players’ actual sharing rates, suggesting that participants misperceived a group difference in sharing (when none actually existed). However, when this subjective misperception was covaried in an analysis of choice behavior, the effect of group stereotype remained significant, *B* = 0.21, *SE* = 0.05, *t* = 3.97, *P* < .001. Thus, the effect of stereotypes on instrumental choice preferences was independent of participants' subjective perception of player reward rates.

The third aim was to determine whether participants could inhibit the influence of stereotypes in their explicit responses, despite the stereotype effect on instrumental learning. Following the main task, Study 3 participants completed a single-round trust game with each player, in which they could entrust a portion of their winnings from the sharing game to a player for a potentially larger return (26; *SI Appendix*). Participants were told that the entrusted amount would be quadrupled, and that the return from each player would be based on that players’ responses in the prior sharing game. Unlike decisions in the choice task, which involved binary classifications made under a 2 s response deadline, trust game decisions involved deliberation about potential payouts, with 10 choice options per round and unlimited decision time. Results showed that participants’ explicit trust decisions reflected only the players’ actual reward rates from the sharing game, with more money entrusted to higher-reward players, *B* = 5.87, *SE* = 1.34, *t*(693) = 4.38, *P* < .001, 95% CI [3.24, 8.50]. Trust decisions were not influenced by group stereotypes, *B* = 0.63, *SE* = 0.42, *t*(693) = 1.48, *P* = .14, 95% CI [−0.19, 1.45], suggesting that the stereotype knowledge was successfully inhibited in explicit responses.

Finally, to ensure that the group effects on choice preferences in Studies 1-3 were not due to wealth cues included in the stereotypes, this procedure was repeated in Study 4 *(N* = 105, preregistered: https://aspredicted.org/RBP_FXD), using stereotype descriptions that omitted references to wealth. Study 4 results replicated those of Studies 1 to 3: Participants’ behavioral choice preferences again reflected group stereotypes (*B* = 0.36, *SE* = 0.04, Wald *z* = 8.46, *P* < .001), in addition to players’ actual reward rates (*B* = 2.29, *SE* = 0.14, Wald *z* = 16.76, *P* < .001), demonstrating that the stereotype effect on instrumental preferences was not due to beliefs about a player’s wealth. Moreover, as in Study 3, participants self-reported a group difference in sharing that did not actually exist, *B* = 4.44, *SE* = 1.34, *t* = 3.32, *P* < .001—a misperception suggesting they believed that their group preference was driven by players’ actual behavior (*SI Appendix*).

Together, Studies 1 to 4 demonstrate that exposure to explicit social stereotypes leads to the formation of internalized group preferences through the process of instrumental learning during interactions with group members. Computational modeling indicated that this pattern reflects the influence of stereotypes on both initial expectancies (priors) and the updating of group member preferences based on reward feedback (leaning rates). This effect of stereotypes on instrumental learning appeared to be implicit; whereas participants inhibited stereotype effects in their explicit decisions, these stereotypes influenced their behavioral preferences independently of their subjective perception.

Having observed the transmission of societal stereotypes to individual-level group preferences in Studies 1 to 4, we next considered a secondary form of transmission, whereby stereotype-based preferences spread to people who merely observe interactions between a stereotype-exposed actor and group member (27). Prior research shows that observers often misattribute an actor’s biased behaviors to qualities of the group member, leading the observer to form their own group bias (27, 28). These findings suggest a pathway through which societal-level stereotypes, once internalized in an individual’s group preferences, may propagate back into a society.

**Fig. 4.**
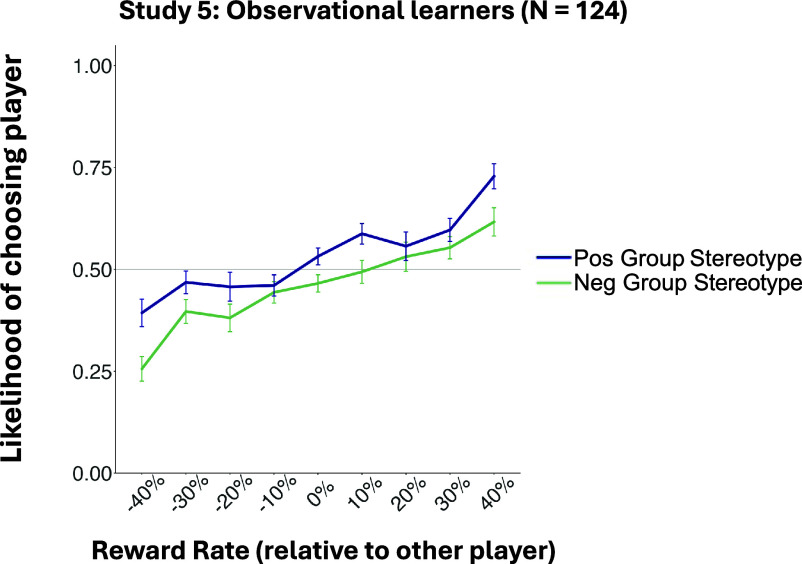
Behavioral choice preferences during the test phase for observational learners in Study 5 as a function of reward rate and group stereotype. Choice preferences of naïve observers reflected the stereotype-biased preferences of demonstrators, in addition to players’ actual reward rates. The x-axis represents the difference between actual reward rates of the two players on a given trial. Error bars indicate SE.

In Study 5 (*N* = 124, preregistered: https://aspredicted.org/STK_EXP), participants played the money-sharing game as in Studies 1 to 4. However, instead of learning directly from group members in a training phase, participants observed the training-phase choices and feedback of a prior participant (demonstrator) across 160 trials. Observers were told they should observe and learn from each player’s feedback to improve their own chances of winning money in a subsequent test phase with the same players. Crucially, observers were not exposed to the stereotype descriptions provided to demonstrators; they were told only that players came from two different groups. Each Study 5 participant (observer) viewed the learning phase interactions of a participant from Study 2, in which a demonstrator made choices and received feedback from players. Two observers were yoked to each Study 2 direct learner. Participants then made their own choices in a test phase (identical to the test phase in Studies 1 to 4). Following the task, participants reported estimated reward rates for each player. This yoked design allowed us to trace the influence of the stereotype message through the direct learner to the group preferences of an observer (Fig. 4).

**Fig. 5.**
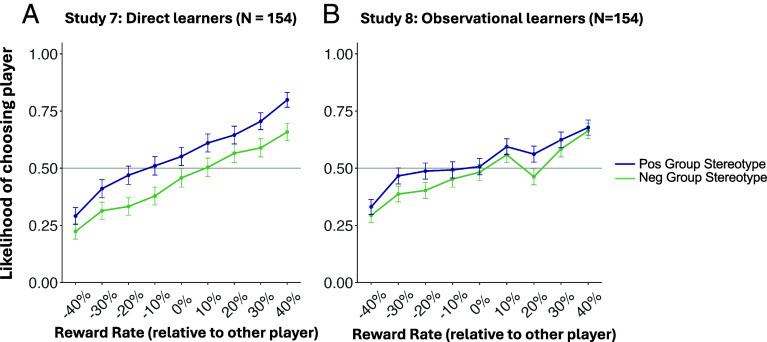
(*A*) Behavioral choice preference for the test phase of Study 7. Participants’ choices reflected the group stereotype, in addition to player reward rates, despite instruction to ignore stereotypes. (*B*) In Study 8, observers naïve to group stereotypes who viewed the learning phase choices and reward feedback of Study 7 participants showed a group bias in their own test phase choice preferences. The x-axis represents the difference between actual reward rates of the two players on a given trial. Error bars indicate SE.

Did the mere observation of demonstrators’ behavior and feedback induce a group preference in observers? It did: observers exhibited a significant group bias in their own test phase choices, despite having no exposure to the stereotype (*B* = 0.32, *SE* = 0.04, Wald *z* = 8.03, *P* < .001), in addition to learning from players’ rewards (*B* = 1.49, *SE* = 0.09, Wald *z* = 16.73, *P* < .001, Fig. 5). Moreover, the magnitude of their group bias correlated with the degree of bias exhibited in the demonstrator’s own test phase choices (*B* = 0.28, SE = 0.09, Wald *z* = 3.21, *P* = .001), indicating that the demonstrator’s degree of prejudice was transmitted to the observer. These findings suggest a cycle of bias propagation, from societal stereotypes to an individual’s group preferences, and then to naïve third-party observers.

Finally, having found that social stereotypes can be internalized in one’s own choice preferences through instrumental learning and propagated to others through observation, we returned to the question we began with: can exposure to societal stereotypes induce internalized group preferences through social-instrumental learning even when people explicitly attempt to ignore the stereotype?

In Study 6 (*N* = 106, https://aspredicted.org/BDH_CDH), participants were exposed to group stereotypes as in Study 4. However, unlike prior studies, these participants were then informed that a) the descriptions were common stereotypes which were unreliable and b) participants should attend only to the feedback of individual players to maximize points. This procedure mimicked the common real-world experience of being exposed to stereotype information but cautioned to ignore it. Nevertheless, despite these instructions, participants’ choice behavior continued to reflect the group stereotypes, *B* = 0.57, *SE* = 0.04, Wald *z* = 13.65, *P* < .001, in addition to players’ actual reward rates, *B* = 2.33, *SE* = 0.13, Wald *z* = 17.50, *P* < .001. Moreover, participants’ self-reports of player sharing rates were predicted by group membership, *B* = 4.83, *SE* = 1.41, *t* = 3.42, *P* < .001, in addition to their actual reward rates, *B* = 46.05, *SE* = 4.46, *t* = 10.33, *P* < .001, again suggesting that the stereotypes led participants to misperceive a difference in group members’ behavior that did not actually exist.

Study 7 (*N* = 154, https://aspredicted.org/V8W_7ZC) repeated the Study 6 procedure with more stringent instructions: After viewing group stereotypes and receiving instructions to individuate, but before beginning the main task, participants completed an understanding quiz. This quiz required participants to correctly indicate their task goal—to choose based on individual player feedback and not group stereotypes—before proceeding to the main task. Despite these explicit instructions and confirmation of participants’ understanding, participants’ choice preferences continued to reflect the stereotype messages (*B* = 0.44, *SE* = 0.04, Wald *z* = 12.50, *P* < .001), in addition to players’ actual rewards (*B* = 2.33, *SE* = 0.11, Wald *z* = 20.90, *P* < .001, Fig. 5*A*). Furthermore, participants’ self-reported estimates of player sharing rates were predicted by group membership (*B* = 1.98, *SE* = 0.18, *t* = 11.20, *P* < .001), in addition to actual reward rates (*B* = 41.02, *SE* = 0.55, *t* = 75.1, *P* < .001). Thus, as in Study 6, participants were unable to prevent the influence of stereotypes on their instrumental learning of group members, and they again misperceived a group difference in player sharing rates that did not actually exist.

In a final study, we tested whether the hypothesized cycle of bias transmission—from societal stereotype to individual to community members—would emerge even when direct learners dismissed the stereotype. In Study 8 (*N* = 154, https://aspredicted.org/H6M_SSZ), participants observed the learning phase trials of Study 7 participants—direct learners who were instructed to ignore the stereotype. Observers, naïve to the stereotype messages, were matched to Study 7 demonstrators in a yoked design (1-to-1 yoking), similar to Study 5. Here again, we found that observers formed group preferences that were consistent with stereotype knowledge of demonstrators (*B* = 0.19, *SE* = 0.03, Wald *z* = 5.67, *P* < .001), in addition to players’ actual reward feedback (*B* = 1.50, *SE* = 0.11, Wald *z* = 13.96, *P* < .001, Fig. 5*B*). The degree of group preference acquired by observers was directly associated with the preference of their respective demonstrator (*B* = 0.15, *SE* = 0.05, *t* = 2.30, *P* = .003). These results demonstrate that stereotype messages can induce a prejudice in direct learners which can then spread to naïve observers, even when the direct learners explicitly attempted to ignore the stereotype.

## Discussion

We asked whether exposure to societal stereotypes can induce personal group-based preferences by shaping the way one learns about group members in direct interactions. Across six studies, we found that positive and negative group stereotypes, conveyed explicitly, shaped the process of instrumental learning in direct interactions with group members. Computational modeling suggested this effect involved the interplay of two processes: Stereotypes set initial expectancies for each group and then influenced the updating of reward values associated with individual group members. This effect of stereotype exposure on instrumental learning appeared to occur implicitly: Although participants were aware of the stereotype content and could inhibit its effect in their explicit trust decisions, they could not prevent its effect on their instrumentally learned preferences toward group members. These findings reveal a mechanism through which mere exposure to stereotype information can bypass an individual’s explicit intentions to induce an internalized group preference.

Next, to examine the broader impact of this mechanism for societal-level prejudice, we asked whether these group choice preferences—formed in response to stereotype exposure—could spread to observers of these interactions via social learning (27, 29). Indeed, in two additional studies, we found that stereotype-induced preferences in participants’ choice behavior were acquired unwittingly by observers who, after viewing this behavior with no knowledge of group stereotypes, expressed stereotype-consistent preferences in their own choices. These findings build on our initial results to illustrate how group preferences produced by stereotype exposure may propagate throughout a community.

This research introduces a model of intergroup bias that describes how exposure to a societal stereotype can induce individual-level prejudice, even among individuals who personally reject the stereotype. Although the importance of considering both individual and societal aspects of intergroup bias is well recognized (30–33), few studies have examined the psychological pathways through which they interact (34). By integrating existing models of stereotyping, based on semantic knowledge representations, with instrumental learning models of direct and observational learning, the present research specifies such a pathway. In doing so, it provides a theoretical framework for understanding how systemic disparities in one’s environment may be internalized in the mind of the individual.

The transmission of societal stereotypes to individual prejudice observed in our studies appeared to occur without participants’ awareness. That is, while participants were aware of the stereotype content and could inhibit its effect on their explicit responses, they appeared unaware of the stereotype influence on the preferences they formed through interaction-based instrumental learning. This effect was likely due, in part, to its indirect nature: Although participants’ explicit goal was to choose players based on individual sharing rates, the task afforded an indirect influence of group membership—much like in real intergroup interactions—which may have been difficult to detect and inhibit. This pattern is further consistent with the nondeclarative operation of instrumental learning which, in past research, has been shown to occur in the absence of awareness (19, 20). These features—the indirect nature of stereotypes on social-interactive instrumental learning and its nondeclarative operation—suggest a potent form of implicit prejudice that has not been previously explored.

A potential alternative account of our findings is that participants simply applied the stereotype knowledge they were given, much like a base rate. However, several aspects of our findings suggest that a “base rate” explanation is unlikely. First, computational modeling across six studies consistently showed that group preferences were explained not just by stereotype priors, but also by stereotype effects on learning; by contrast, a base rate model in which preferences were determined by stereotype priors without learning (the “stereotype only” model) was the worst-fitting model. Second, participants formed group preferences even when the stereotype was explicitly discounted and they were instructed to ignore it, and despite financial incentives opposing the stereotype. And third, participants reported perceiving a group difference in sharing despite equated reward rates, further suggesting that participants’ group preferences reflected their direct learning experiences and not merely the application of a base rate.

Our research contributes methodological advances to the study of intergroup bias through its use of computational modeling to systematically test and compare theories of stereotype function. Here, we adapted models of rule-based priors on RL (21, 35) to address the effect of stereotype knowledge on interactive learning (36). By formalizing and comparing alternative models, we found strong support for the hypothesized *stereotype learning model*, whereby stereotypes operated as priors and differentially affected learning from group members. This approach complements prior research on biased sampling in the formation of prejudice (37–39), further illustrating how computational modeling may be used fruitfully to investigate mechanisms of social cognition and their interplay with features of society (27, 40–44).

More broadly, our findings show that messages promoting societal stereotypes are more than mere words; exposure to biased group descriptions can shape one’s subsequent experiences with members of the group, perhaps without one’s knowledge, in ways that confirm the message and spread it to others. This process—whereby societal stereotypes are transmitted to personal group preferences—may also help to explain how systemic biases, such as institutional inequality, may be transmitted via stereotypes from social structures to the minds of individuals (45–48). As society continues to grasp the impact of polarizing sociopolitical rhetoric, from campaign ads to social media, our findings suggest that its influence may be more potent and far-reaching than previously thought. Yet, by illuminating the processes through which explicit societal messages may induce personal bias in the individual, these results may inform approaches to reducing their impact.

## Materials and Methods

### Ethics.

Ethics approval was obtained from the human subjects institutional review boards at the University of Amsterdam and New York University. All participants provided informed consent prior to their participation.

### Stereotype Manipulation.

Participants learned that they would play a money-sharing game with players from two social groups. Before beginning the task, participants were given the following descriptions of these groups (counterbalanced across participants):

*“In the main task you will play an interactive money-sharing game with people from two different groups who come from different places. For the purpose of this study, we will refer to these groups as Group A and Group B, and their members will be represented by avatars. Members of Group A live in a more affluent society, where crime is low and most people have good jobs. People from Group A are often perceived to be trustworthy, honest, and generous to others, and they are proud of their success. Group B, by comparison, lives in a society that is economically poor, with a high rate of unemployment and serious crimes such as robbery, assault, and murder. People from Group B are often perceived to be hostile, untrustworthy, and dishonest.”*

Participants were then shown avatars representing players from each group, with color cues (blue vs. green clothing, darker vs. lighter hair) signaling group membership. Participants interacted with either all female or all male-appearing avatars. Participants were instructed that players had participated in a previous experiment in which they decided how many points (redeemable for a monetary bonus) to share. Participants were further told that different players shared different amounts, and they should learn who shared more often to win the most points.

### Sharing Game.

The main learning task, presented as a sharing game, consisted of a 160-trial training phase and a 96-trial test phase. In the training phase, participants always chose between two targets—one from each group—with fixed complementary reward probabilities (70% vs. 30% or 60% vs. 40%). Although the reward feedback varied within groups, there was no difference between groups. On each trial, a face pair was shown for a maximum of 2 s, during which time a response was required. Reward feedback (+1 or 0 points) appeared immediately following choice. Player gender and group color cue (blue or green) were counterbalanced and the assignment of player identity to reward rate was randomized across participants.

The test phase provided a readout of learned reward values. Participants chose between all combinations of targets from different groups, always with one Group A member and one Group B member. Each pair was shown for a maximum of 2 s, during which time a response was required, followed by a 1,000 ms intertrial interval. Feedback was not given, to prevent further learning, but participants received a cash bonus for choosing high-sharing players which was paid after task completion.

### Computational Modeling.

Computational RL models used to evaluate our hypothesis and alternatives were based on the standard Q-learning rule:

$${Q_{i}}^{t+1}={Q_{i}}^{t}+\alpha (R^{t}-{Q_{i}}^{t})$$

where *Q_i_* is the action value of selecting option *i* in trial *t*, *R* is the reinforcement [no reward = 0, reward = 1] received in trial *t*, and *α* (0 ≤ *α* ≤ 1) is a learning rate parameter, which determines how much the difference between the received and the predicted reinforcement (the prediction error) affects subsequent value estimates

These Q-values were then transformed into decision probabilities using a standard Softmax function:

$$P_{i}=\frac{e^{Q_{i}/\beta }}{\sum_{j=1}^{2}e^{Q_{j}/\beta }}$$

To examine effects of group-based initial expectations, the model was formulated using a symmetrical prior parameter (ranging from −100 to +100):

$$Q_{\mathrm{Good}}^{t=0}=prior,Q_{\mathrm{Bad}}^{t=0}=-prior$$

To examine effects of target group on learning, models included separate learning rates as a function of group membership:

$${Q_{i,group}}^{t+1}={Q_{i,group}}^{t}+\alpha_{\mathrm{group}}(R^{t}-{Q_{i,group}}^{t})$$

Detailed descriptions of methods may be found in *SI Appendix*.

## Supplementary Material

Appendix 01 (PDF)

## Data Availability

Anonymized behavioral data will be deposited in OSF (49).
