# Supplementary material for: Transmission of societal stereotypes to individual-level prejudice through instrumental learning
Source: Proc Natl Acad Sci U S A. 2024 Nov 1;121(45):e2414518121. doi: 10.1073/pnas.2414518121 (PMC11551433; doi:10.1073/pnas.2414518121)
Supplement: Supplementary file 1 — Appendix 01 (PDF) [file pnas.2414518121.sapp.pdf]

PNAS

Supporting Information for

Transmission of societal stereotypes to individual-level prejudice through instrumental learning

David T. Schultner[1†] & Benjamin S. Stillerman[2†], Björn R. Lindström[3], Leor M. Hackel[4], Damaris R. Hagen[1], Nils B. Jostmann[1], & David M. Amodio[1*]

[†]Shared first authorship

[1]University of Amsterdam
[2]New York University
[3]Karolinska Institutet
[4]University of Southern California

*Corresponding author: David M. Amodio
Email: david.amodio@gmail.com

This PDF file includes:

Supporting text
Figures S1 to S4
Tables S1 to S2
SI References

Table of Contents

Study 1 **3**
Method 3
Results 7
Study 2 **8**
Method 8
Results 9
Study 3 **10**
Method 10
Results 12
Study 4 **14**
Method 14
Results 15
Study 5 **16**
Method 17
Results 19
Study 6 **21**
Method 22
Results 23
Study 7 **24**
Method 24
Results 25
Study 8 **26**
Method 26
Results 26
Computational modeling **27**
Supporting Information References **36**

Study 1

Overview. In Study 1, we tested whether explicit descriptions of groups would bias participants' choices of who to interact with and win money from. Participants read descriptions of two fictional groups and then played an economic game with ostensible players from those groups. Group membership was, on average, not associated with reward probability and thus not beneficial cue for choice performance.

Method

Participants

Sixty-nine students at University of Amsterdam received course credit for their participation as well as a performance-based monetary bonus, ranging from $1.30 – $1.70. In this and subsequent reported studies, we excluded participants who failed to reach a learning criterion of 50% accuracy for 30%-70% player pairs during the test phase (i.e., A-B and G-H; see below for details of test phase procedure). In Study 1, this exclusion criterion yielded a final sample size of N = 61 (45 women, 16 men; M_{age} = 21.56 years, SD_{age} = 5.20 years).

Ethics approval was obtained from the human subjects institutional review board at the University of Amsterdam.

Procedure

Introduction and manipulation. Upon arrival to the lab and following informed consent, participants learned that they would play a money sharing game with players from two social-geographical groups. Before beginning the task, participants were given the following descriptions of these groups (with descriptions of Group A and B counterbalanced across participants):

> "In the main task you will play an interactive money-sharing game with people from two different groups who come from different places. For the purpose of this study, we will refer to these groups as Group A and Group B, and their members will be represented by avatars. Members of Group A live in a more affluent society, where crime is low and most people have good jobs. People from Group A are often perceived to be trustworthy, honest, and generous to others, and they are proud of their success. Group B, by comparison, lives in a society that is economically poor, with a high rate of unemployment and serious crimes such as robbery, assault, and murder. People from Group B are often perceived to be hostile, untrustworthy, and dishonest."

These descriptions were based on common societal stereotypes of White and Black Americans, respectively (1), and which also correspond to common stereotypes to White (native) and Moroccan Dutch immigrants.

This stereotype information was followed by a note that, despite these generalizations, there is individual variability, and that the participant should pay attention to individual players' behavior:

> "So, as you see, these groups are different in many ways. However, individuals within each group vary, too. You will need to learn about these people as you engage in repeated interactions in the task."

Participants were then shown avatars representing players from each group, with color cues (blue vs. green clothing, darker vs. lighter hair) signaling group membership (all other features were matched between groups). Participants were assigned, in counterbalanced fashion, to view either all female or all male-appearing avatars (Figure S1), to control for potential target gender effects. Participants were instructed that these players had participated in a previous experiment in which they decided how many points (redeemable for a monetary bonus) to share. Participants were further told that different players shared different amounts, and they should learn who shared more often to win the most points.

Categorization task. To ensure that participants learned the group identity of each player, they completed a categorization task embedded in a standard 7-block implicit association test (IAT). The first and fifth blocks of this IAT required simple categorizations of player to their group category, with accuracy feedback. This IAT was repeated at the very end of the task. Although not the focus of these studies, Time 1 IAT data indicated that explicit group descriptions alone created a significant IAT effect, with preference expressed toward Group A, similar to much prior research (e.g., 2), showing that IAT scores can be driven by a variety of influences including novel explicit group beliefs. The same pattern of Group A preference was observed at Time 2. We did not analyze IAT scores further, and the measure was dropped from all subsequent studies.

Figure S1. Sample avatars and schematic of reward probabilities.

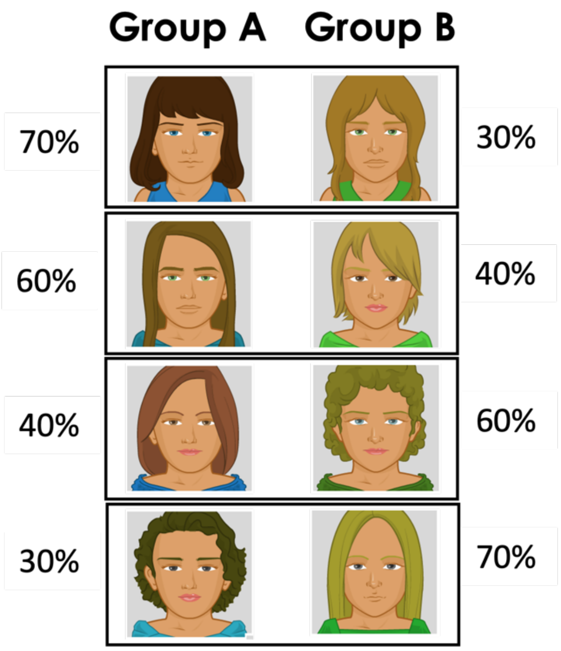

Learning task. Next, participants completed the main learning task, which included a training phase of 160 trials and a test phase of 96 trials. In the training phase, participants always chose between two targets—one from each group—with complementary fixed reward probabilities (e.g., player pairs A-B, C-D, E-F, and G-H, see Figure S1). On each trial, a face pair was shown for a maximum of 2 s, during which time a response was required. Reward feedback (+1 or 0 points) appeared immediately following choice, and points were converted to a cash bonus at the task conclusion. The cover story was that this feedback was derived from past participants' actual choices of how often to share points. Crucially, although the reward feedback varied by individuals within groups, average reward rate for both groups was equated. Player gender and group color cue (blue or green) were counterbalanced across participants, and player identity was randomized such that individual players were assigned to random reward rates for a given participant. To win as much money as possible, participants were motivated to learn which target players tended to reward more often than others.

Next, in the test phase, in order to obtain a readout of learned reward values, participants chose between all combinations of targets from different groups (e.g., A-B, A-D, A-F, A-H, C-B, etc.), always with one Group A member and one Group B member. Each pair was shown for a maximum of 2 s, during which time a response was required, followed by a 1000 ms intertrial interval. Feedback was not given, to prevent further learning, but participants were told that correct choices would still be rewarded and paid out in the bonus at the end of the task.

Results

Primary analysis focused on test phase data and involved two approaches: multilevel regression and computational modeling. We detail the regression approach below and, for all studies, report computational modeling results in the section “Computational modeling”.

Multilevel regression was used to test effects of (a) players’ actual reward rate and (b) group membership on choice. Trials in which choices were made faster that 200 ms or slower than 2000 ms were excluded from analysis. Participants’ trial-level choice data were submitted to a general linear mixed model predicting the likelihood that participants chose a given target player, nested by participant, with a logit link function. Specifically, we predicted the probability of choosing the left target as a function of (a) its reward rate relative to that of the right target and (b) its group membership. The primary model included by-participant random intercepts and the following predictors as fixed effects: players’ actual reward rate, players’ group membership, and their interaction. For completeness, we also report models with by-participant random slopes for the fixed effects. Sharing rates were equated between groups (t-test for a group difference: t = -0.45, df = 9505, p = 0.66).

Results indicated a significant effect of player’s relative reward rate on choice, demonstrating learning of player reward rates, B = 2.68, SE = 0.19, Wald z = 14.43, p < .001. An examination of raw choice behavior revealed a relatively accurate mapping between participants’ choices and the actual reward contingencies. Importantly, the effect of group membership on choice was also significant, such that participants were more likely to choose Group A members over Group B members, B = 0.52, SE = 0.06, Wald z = 9.33, p < .001. Indeed, when faced with two equally rewarding players, participants chose the Group A member 25%

more often. The Reward Rate x Group interaction was not significant, *B* = -0.003, SE = 0.26, Wald *z* = -0.01, *p* = .992. The pattern was qualitatively identical in the random slopes model (Reward rates: *B* = 3.27, SE = 0.36, Wald *z* = 9.08, *p* < .001, Group bias: *B* = 0.65, SE = 0.23, Wald *z* = 2.79, *p* = .005)

To corroborate these findings, training phase data were submitted to the same general linear mixed model. Results replicated those of the test phase data, with evidence of accurate learning of player reward rates, *B* = 1.77, *SE* = 0.10, Wald *z* = 18.43, *p* < .001, as well as a bias to choose Group A, *B* = 0.29, *SE* = 0.04, Wald *z* = 6.95, *p* < .001. The reward rate by group interaction was marginally significant, *B* = -0.25, *SE* = 0.13, Wald *z* = -1.83, *p* = .068.

Study 2

Overview. In Study 2, we sought to replicate the findings of Study 1 in a new sample. The procedure was identical to that of Study 1, except that the initial categorization task and IATs were dropped and it was conducted online rather than in the lab.

Ethics approval was obtained from the human subjects institutional review board at New York University.

Method

Participants. Participants were 78 Amazon Mechanical Turk (MTurk) workers (demographics unavailable due to technical error) who received $2.00 for their participation as well as a performance-based monetary bonus, ranging from approximately $0.30 – $0.40, derived from points earned during the task with a conversion rate of 2 points per cent.

Participants who failed to reach a learning criterion of 50% accuracy for choices between 30% vs. 70% reward player pairs during the test phase (i.e., A-B and G-H; N = 16) were excluded. After exclusions, Study 2 had a final sample size of N = 62.

Procedure. Participants completed an online learning task, nearly identical to the one described in Study 1. Besides a slightly different look and feel for the online version, the only difference was that the groups described with positive and negative stereotypes were counterbalanced (e.g., whether Group A or Group B was described as the good group). For ease of reporting and visualizing the analysis, we refer to the group described with positive and negative stereotypes as “Group A” and “Group B,” respectively, in the results.

Results

Our analytical approach followed that of Study 1, with a focus on test phase choice data. Results indicated significant effects for players' actual reward rate, demonstrating strong learning, B = 2.55, SE = 0.19, Wald z = 13.51, p < .001, and for group membership, such that participants strongly preferred Group A members independent of actual reward rates, B = 0.79, SE = 0.06, Wald z = 13.86, p < .001. The Reward Rate x Group Membership interaction was not significant, B = -0.35, SE = 0.26, Wald z = -1.32, p = .187. As in Study 1, the qualitative pattern was identical in the random slopes model Reward rates: B = 3.03, SE = 0.39, Wald z = 7.7, p < .001, Group bias: B = 1.02, SE = 0.37, Wald z = 2.8, p = .005)

As in Study 1, to corroborate these findings, we submitted the training phase data to the same general linear mixed model. The results replicated those of the test phase data, with significant effects of actual reward rate, B = 1.40, SE = 0.10, Wald z = 14.66, p < .001, and of

group membership, evidencing a preference for Group A members, *B* = 0.45, *SE* = 0.04, Wald *z* = 10.59, *p* < .001. The interaction was not significant, *B* = 0.17, *SE* = 0.14, Wald *z* = 1.24, *p* = .214.

Study 3

Overview. In Study 3, we extended the procedure used in Studies 1 and 2 to include two additional post-learning-task measures: explicit beliefs of player reward rates and a trust game.

Method

Participants. Participants were 158 Amazon Mechanical Turk (MTurk) workers who received $2.00 for their participation as well as a performance-based monetary bonus, ranging from approximately $0.30 – $0.40, derived from points earned during the task with a conversion rate of 2 points per cent. We excluded participants who responded without variation for either of the post-learning-task measures (*N* = 18) and participants who failed to reach a learning criterion of 50% accuracy for 30%-70% player pairs during the test phase (*N* = 47). One participant was excluded due to a technical error resulting in invalid post-learning-task measures. Our exclusion of trials with invalid reaction times resulted in 5 participants being excluded altogether. These exclusions resulted in a final sample size of *N* = 87 (44 men, 36 women, 7 unreported; M_{age} = 34.9 years, SD_{age} = 10.0 years).

Ethics approval was obtained from the human subjects institutional review board at New York University.

Procedure. After reading the group instructions, participants completed a categorization task to reinforce the group membership of target players. Unlike the categorization task used in Study 1, which was embedded within an IAT, Study 3 used a stand-alone task that included the

classification of both group member faces and trait terms that had been conveyed in the group description manipulation. Hence, participants were presented with pictures of players and stereotype words associated with the group descriptions (e.g., “wealthy,” “uneducated,” “trustworthy”) and classified each according to group label. They then completed the learning task, as in Study 2; however, in Study 3, Group A always associated with the positive stereotype description and Group B was associated with the negative stereotype description. This decision was made because A and B are often associated with better and worse options, respectively, and this could contribute to noise or confusion with the manipulation.

After the learning task, participants completed a subjective reward measure, in which they were asked, for each player in randomized order, “How many times out of a hundred would this player share with you?” For each player, participants typed their estimate of the player’s sharing rate, from 1 to 100, in a text box. This form of response was designed to assess declarative semantic knowledge, which might be expressed independent of any striatally-based instrumental tendencies that could influence responses on a slider-type scale (Knowlton et al. 1996).

Finally, participants played a single-shot trust game with each target player. They were told they had a 20-point pool and they could choose how much to invest in each player as a trustee. The trustee’s point amount would then be tripled and they could share any amount back to the participant. For each player, the participant selected a number of points to share, from 0 to 20, with options at 2-point intervals. Unlike the sharing game, which permitted a maximum of 2 seconds for binary decisions, the trust game allowed for deliberate choices with unlimited time and ten answer options per round.

Results

Choice behavior. Our analytical approach followed that of Studies 1 and 2. Multilevel regression predicting player choice again produced significant effects of players' actual reward rate, B = 1.76, SE = 0.16, Wald z = 11.15, $p < .001$, as well as group membership, with participants preferring Group A members, B = 0.48, SE = 0.05, Wald z = 9.58, $p < .001$. Again, the interaction was not significant, B = 0.01, SE = 0.23 Wald z = 0.05, p = .964. The random slopes model produced the same pattern (Reward rates: B = 2.01, SE = 0.31, Wald z = 6.5, $p < .001$, Group bias: B = 0.52, SE = 0.19, Wald z = 2.74, p = .006)

As in Studies 1 and 2, analysis of training phase data produced the same pattern: significant effects of players' reward rate, B = 0.93, SE = 0.08, Wald z = 11.04, $p < .001$, and of group membership, B = 0.46, SE = 0.04, Wald z = 12.09, $p < .001$, on choice. The interaction was not significant, B = 0.04, SE = 0.12, Wald z = 0.40, p = .691.

Subjective rewards. Participants' subjective reward estimates were submitted to a linear regression, with actual player reward rate and player group as predictors. Subjective estimates were significantly predicted by players' actual reward rates, B = 31.31, SE = 8.49, t = 3.69, $p <$ .001, indicating participants had some knowledge of the reward contingencies (Figure 2A). Subjective reward rates were also predicted by group membership, B = 3.88, SE = 1.90, $t(683)$ = 2.04, p = .042, suggesting a weak effect of group bias on subjective reward in addition to the relatively strong effect effects observed on choice behavior.

Figure S2. (A) Estimated reward rates for each player. Study 3 participants estimated higher reward rates for more rewarding players and for Group A members. (B) Number of points entrusted to players during the trust game. Study 3 participants entrusted more points to the more rewarding players and to Group A members.

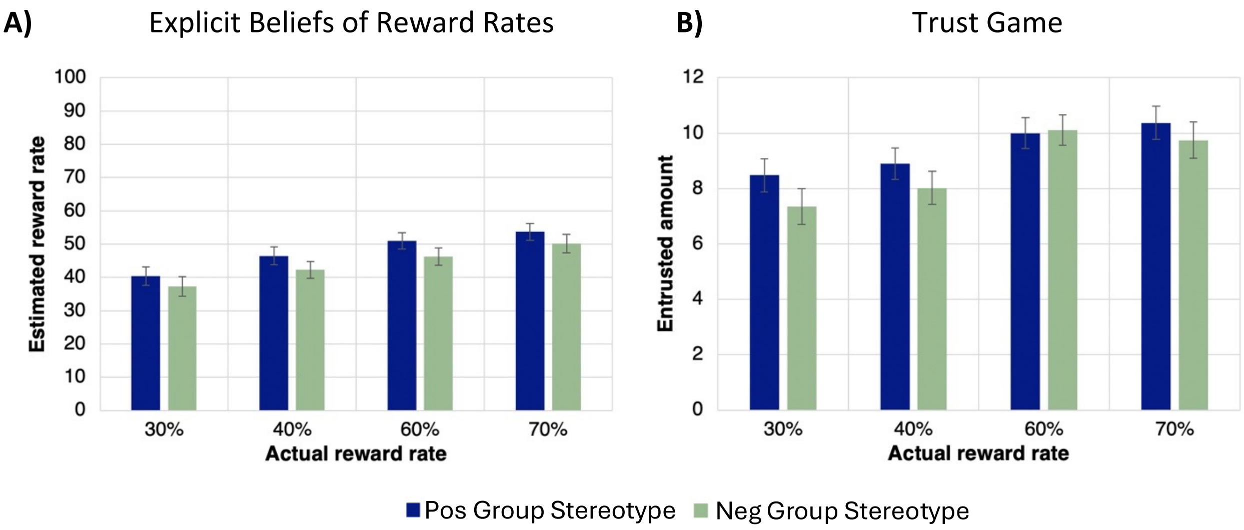

To test whether the effects of group membership on choice behavior could be explained by subjective rewards, or whether the group bias instrumental learning operated independently of subjective rewards, we conducted an analysis in which player reward rates, group membership, and subjective estimates were each included in the main multilevel model predicting test phase choice. We found that while subjective rewards predicted choices to a small extent, B = 0.03, SE = 0.01, Wald z = 26.62, p < .001, actual reward rates (B = 0.94, SE = 0.11, Wald z = 5.44, p < .001) and player group (B = 0.21, SE = 0.05, Wald z = 3.97, p < .001) remained strong predictors of choice behavior. This result suggests that subjective beliefs about group differences in reward did not fully account for the group effect expressed in behavior.

Trust game behavior. Participants' trust game investments were submitted to a linear regression, with players' (trustee) true reward rate and player (trustee) group as predictors.

Participants' investments were significantly predicted by players' actual reward rates, $B = 5.87$, $SE = 1.34$, $t(693) = 4.38$, $p < .001$, 95% CI [3.24, 8.50], reflecting that reward learning translated to an expression of trust (Figure 2B). However, the effect of group membership was not significant, $B = 0.63$, $SE = 0.42$, $t(693) = 1.48$, $p = .140$, 95% CI [-0.19, 1.45].

Study 4

Overview. Despite Studies 1-3 consistently showing a transmission of stereotypes to personal preferences, one alternative explanation could be that the stereotype messages provided payoff-relevant information instead of merely creating a generalized positive or negative portrayal. That is, since parts of the stereotypes alluded to differences in wealth levels between the social groups (groups were described as coming from either more or less affluent regions with low or high unemployment), these descriptions may have implicitly communicated information about the expected sharing rates of both groups. In Study 4 (preregistered: https://aspredicted.org/RBP_FXD), we modified the stereotype descriptions used in Studies 1-3 to exclude any wealth-related information.

Method

Participants. Participants were 134 workers on the recruitment platform Connect who received $4.00 for their participation as well as a performance-based monetary bonus, ranging from $0 – $3.00, derived from points earned during the task. We excluded participants who failed to reach a learning criterion of 50% accuracy for 30%-70% player pairs during the test phase, as well as participants who did not finish the main task ($N = 29$). These exclusions

resulted in a final sample size of *N* = 105 (60 men, 41 women, 1 nonconforming, 3 unreported; M_{age} = 39.50 years, SD_{age} = 12.36 years).

Ethics approval was obtained from the human subjects institutional review board at the University of Amsterdam.

Procedure. The procedure was equivalent to that of previous studies, but the stereotype messages now did not include wealth-related cues. The new stereotype messages were:

Members of Group A live in a secure region, where crime is low and which is commonly seen as peaceful. People from Group A are often perceived to be trustworthy, honest and polite. Members of Group B, in contrast, live in a different region which is considered more dangerous, with high rates of serious crimes such as robbery, assault, and murder. People from Group B are often perceived as hostile, untrustworthy, and dishonest.

Results

Choice behavior. Our analytical approach followed that of previous studies. Multilevel regression predicting player choice again produced significant effects of players' actual reward rate, *B* = 2.29, SE = 0.14, Wald *z* = 16.76, *p* < .001, as well as group membership, with participants preferring Group A members, *B* = 0.36, *SE* = 0.04, Wald *z* = 8.46, *p* < .001 (Figure S3). The random slopes model did not show a significant group effect, *B* = 0.49, SE = 0.31, Wald *z* = 1.57, *p* = .12.

Figure S3. Choice behavior for the test phase of Study 4. Participants' choices (solid lines) demonstrated learning of reward contingencies as well as a group bias. Reward rate, displayed on the x axis, represents the actual reward rate of a given player minus the actual reward rate of the alternative player in a trial. Error bars indicate standard error.

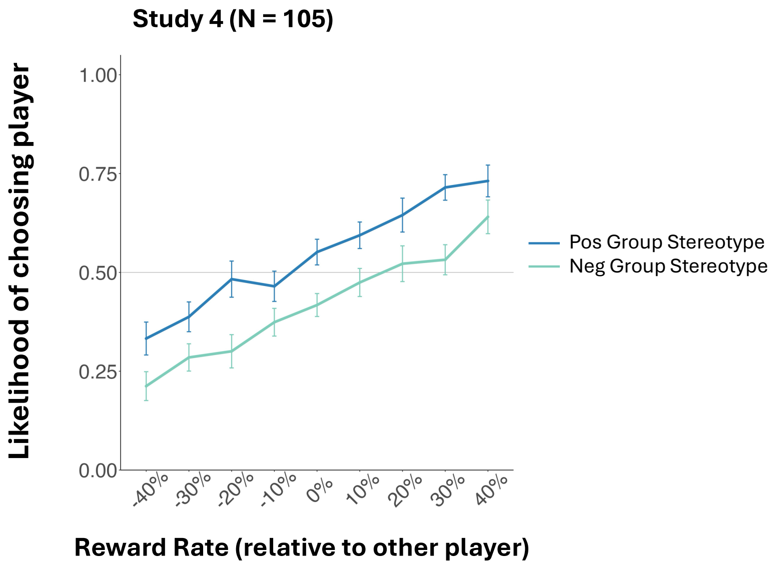

Explicit rewards. A regression with participants' explicit reward ratings and actual reward rates, as well as group membership shows effects for both factors: Reported rewards tracked actual rewards, $B = 43.44$, $SE = 4.22$, $t = 10.29$, $p < .001$, but were also influenced by group, $B = 4.44$, $SE = 1.34$, $t = 3.32$, $p < .001$. Adjusting for reported rewards again retains the group effect in the main analysis, $B = 0.43$, $SE = 0.03$, $t = 16.6$, $p < .001$.

Study 5

Overview. In Study 5, we asked whether the prejudice could be propagated through mere observation of biased choices without any knowledge of group descriptions or direct feedback from group members. In this study procedure, participants observed the training data

of a past participant, viewing their choice and feedback but not being exposed to any group descriptions, and then completed their own test phase choices. Each Study 5 participant was yoked to real Study 2 participant ("demonstrator") and observed the training phase behavior of the participant to whom they were yoked. To test whether participants formed a group bias based on this observational learning, Study 5 participants then made their own choices in the test phase. After the test phase of the learning task, participants reported their estimates of player reward rates in the same explicit belief measure as Study 3, followed by estimates of the choice behavior of the demonstrators that they observed. Study 5 was preregistered at https://aspredicted.org/blind.php?x=6zi6fz.

Method

Participants. Participants were recruited from the online NYU subject pool and received course credit for their participation as well as the chance to win a performance-based monetary bonus of $15 to be awarded to the five top performers. Our stopping rule was to collect data until two Study 5 participants were yoked to each of the 62 demonstrators from Study 2 (for a total N of 124). We excluded participants who responded without variation in the post-learning task (*N* = 6), participants who failed to reach 50% on either attention check measure (*N* = 13; see Procedure), and participants who failed to reach a learning criterion of 50% accuracy for the 30%-70% pairs during the test phase (i.e., A-B and G-H; *N* = 33). Because we could not know the number of eventual exclusions during the period of data collection, data were collected from an additional 36 participants who met inclusion criteria but were ultimately not needed and thus excluded. These extra participants were excluded based on chronological order of completing the experiment, and their data were never analyzed. After all exclusions, this approach yielded

the target final sample size of *N* = 124 (82 women, 39 men, 3 unreported; M_{age} = 19.5 years, SD_{age} = 1.35 years).

Ethics approval was obtained from the human subjects institutional review board at New York University.

Procedure. Participants read instructions similar to Studies 1 – 3, which explained the nature of the sharing task and the fact that players represented two different groups, but they received no descriptions of the groups. They were also told that they were to learn about the target players by watching past “demonstrators” make decisions and receive feedback. Participants then completed a categorization task of the group membership of target players, which served to both reinforce group membership cues and provide an attention check on which to base participant exclusions. Unlike in Study 3, this categorization task did not include stereotype words; participants only categorized faces of group members. Participants with less than 50% accuracy on the classification task were excluded (*N* = 9).

Next, participants observed the training phase, where, instead of making choices, participants observed the trials of the Study 2 demonstrator to whom they were yoked. Trials were presented in the same order and each yoked trial was animated in real time (using actual reaction times for each prior demonstrator choice) to show choices and subsequent reward feedback, identical to how direct learners viewed choices and feedback. Participants observed the entirety of the yoked training phase, complete with a break between the two blocks, with the exception that trials were skipped if they had originally been excluded in Study 2 based on reaction time. To ensure participants paid attention, “catch” trials appeared after some trials, prompting participants to indicate what choice they had just observed on the previous trial.

Twenty catch trials appeared in the observational training phase, occurring in a fixed, pseudorandom order. Participants with less than 50% accuracy on the catch trials were excluded ($N = 4$).

Participants then completed the test phase, making their own choices, as in Studies 1 – 4. Next, participants reported their explicit beliefs about player reward rates, as in Study 3, typing their response in a box under displays of each player ("How many times out of a hundred would this player share with you?"). Finally, participants reported their estimates of demonstrators' tendency to choose each player ("How many times out of a hundred did the Decider choose this player?").

Results

Observational learning effects. Our analytical approach followed thar of Studies 1 – 4, with a focus on test phase choices. In this study, however, participants did not directly complete a training phase, but instead observed training phase behavior of Study 2 participants. As in the previous studies, in which learning occurred directly, multilevel regression indicated that observational learning produced a significant effect of actual reward rates, $B = 1.49$, $SE = 0.09$, Wald $z = 16.73$, $p < .001$, as well as a significant effect of group membership, with Study 5 participants preferring Group A players, $B = 0.32$, $SE = 0.04$, Wald $z = 8.03$, $p < .001$. The interaction was not significant, $B = -0.24$, $SE = 0.18$ Wald $z = -1.36$, $p = .173$. As in the preceding experiments, the random effects analysis produced a qualitatively identical pattern (Reward rates: $B = 1.78$, SE = 0.22, Wald $z = 7.99$, $p < .001$, Group bias: $B = 0.37$, SE = 0.19, Wald $z = 2.0$, $p = .045$). This group bias remained after adjusting for Study 2 participants'

subjective estimates of player reward rates (*B* = 0.26, *SE* = 0.04, Wald *z* = 6.52, $p < .001$), consistent with an implicit transmission of bias.

It should be noted that this analysis deviated slightly from our preregistered plan, which was to yoke Study 5 participants to the total Study 2 sample (*N* = 78), with two Study 5 participants yoked to each Study 2 participant in order to increase power. This pre-registration did not consider that some Study 2 participants would provide invalid or incomplete date. Hence, in order to obtain validity and rigor, Study 5 participants were yoked only to Study 2 participants included in the final Study 2 analysis. Nevertheless, results were nearly identical using this sample (*N* = 156): test phase learning effect: *B* = 1.49, *SE* = 0.11, Wald *z* = 13.36, $p < .001$; group effect: *B* = 0.39, *SE* = 0.03, Wald *z* = 11.25, $p < .001$; interaction: *B* = -0.17, *SE* = 0.18 Wald *z* = -1.13, *p* = .258.

In addition to these analyses, we also estimated the direct transmission of bias by predicting the participants preference for Group A from the demonstrator´s Group A bias using multilevel regression (*B* = 0.28, SE = 0.09, Wald *z* = 3.21, *p* = .001). This result indicates that the degree of demonstrator group preference was significantly correlated was with the degree of observer group preference.

Explicit beliefs. In the reward estimation task, participants' estimates of targets players' reward rates was not significantly associated with those players' actual reward rates, *B* = -2.48, *SE* = 4.99, $t(989) = -0.50$, *p* = .619, indicating participants had very poor, if any, declarative knowledge of the reward contingencies. There was also no evidence of a group bias in estimations of reward rates, *B* = 0.79, *SE* = 1.58, $t(107) = 0.50$, *p* = .615. Thus, the observational

learning of bias appeared to emerge in the absence of explicit beliefs or knowledge regarding player reward rates.

As in Study 3, to more directly test whether choice behaviors reflected a group bias in the absence of explicit beliefs, we tested the main regression with actual player reward rate, group membership, and explicit belief estimates as predictors. Results indicated a small-effect size association between explicit beliefs and choice behavior, $B = 0.01$, $SE = 0.001$, Wald $z = 20.22$, $p < .001$, significant effects remained for actual reward rates, $B = 1.69$, $SE = 0.13$, Wald $z = 13.09$, $p < .001$, and group membership, $B = 0.26$, $SE = 0.04$, Wald $z = 6.52$, $p < .001$.

An analysis of observers' estimate of demonstrator choices indicated that these did not significantly reflect the actual player reward rates, $B = -0.66$, $SE = 4.77$, $t(989) = -0.14$, $p = .890$, or group membership, $B = 1.05$ $SE = 1.51$, $t(989) = 0.70$, $p = .486$.

Study 6

Overview. Previous studies showed that stereotype messages influenced subsequent learning from interactions. To conduct a stronger test of whether stereotype messages, once encoded in memory but not necessarily endorsed, will influence instrumental learning, stereotypes in Study 6 were presented in a context which casts doubts on their veracity. Do stereotype messages affect recipients even if their validity is questioned directly? To answer this question, in Study 6 participants again received group stereotypes, but were subsequently informed that these are merely common stereotype messages which may or may not be true, that individuals within groups vary, and that as a consequence participants should attend to

individual player feedback instead of group stereotypes. Study 6 was preregistered at https://aspredicted.org/BDH_CDH.

Method

Participants. Participants were 148 workers on the recruitment platform Connect who received $4.00 for their participation as well as a performance-based monetary bonus, ranging from $0 – $3.00, derived from points earned during the task. We excluded participants who failed to reach a learning criterion of 50% accuracy for 30%-70% player pairs during the test phase, as well as participants who did not finish the main task (N = 42). These exclusions resulted in a final sample size of N = 105 (50 men, 44 women, 2 nonconforming, 10 unreported; M_{age} = 37.46 years, SD_{age} = 11.17 years).

Ethics approval was obtained from the human subjects institutional review board at the University of Amsterdam.

Procedure. The procedure was equivalent to that of Study 4, but now the stereotypes appeared together with messages questioning their veracity and usefulness:

"In this study, we are interested in how background information, about people's social groups and where they come from, informs our judgments. Although these groups will be kept anonymous, below are descriptions of how each group is typically viewed:

Members of Group A live in a region typically viewed as secure and with low crime, and it is commonly seen as a peaceful place. People from Group A are often perceived to be trustworthy, honest, and polite.

Members of Group B, by comparison, live in a region considered more dangerous, viewed as having high rates of serious crimes such as robbery, assault, and murder. People from Group B are often perceived as hostile, untrustworthy, and dishonest.

As you see, these two groups are perceived to differ in many ways. However, these descriptions are common stereotypes about these groups and may not be true. Individuals in each group

vary, too, and so the stereotypes can often be misleading. It's important that you learn about the individual players as you engage in repeated interactions in the task."

Results

Choice behavior. Our analytical approach followed that of previous studies. Multilevel regression predicting player choice again produced significant effects of players' actual reward rate, $B = 2.33$, SE = 0.13, Wald $z = 17.50$, $p < .001$, as well as group membership, with participants preferring Group A members, $B = 0.57$, $SE = 0.04$, Wald $z = 13.65$, $p < .001$ (Figure S4). The random slopes model showed a significant group effect, $B = 0.86$, SE = 0.29, Wald $z = 2.95$, $p = .0032$.

Figure S4. Choice behavior for the test phase of Study 6. Participants' choices (solid lines) were predicted by rewards and target group membership. The x-axis represents the difference between the reward rates of the two available players on a given trial.

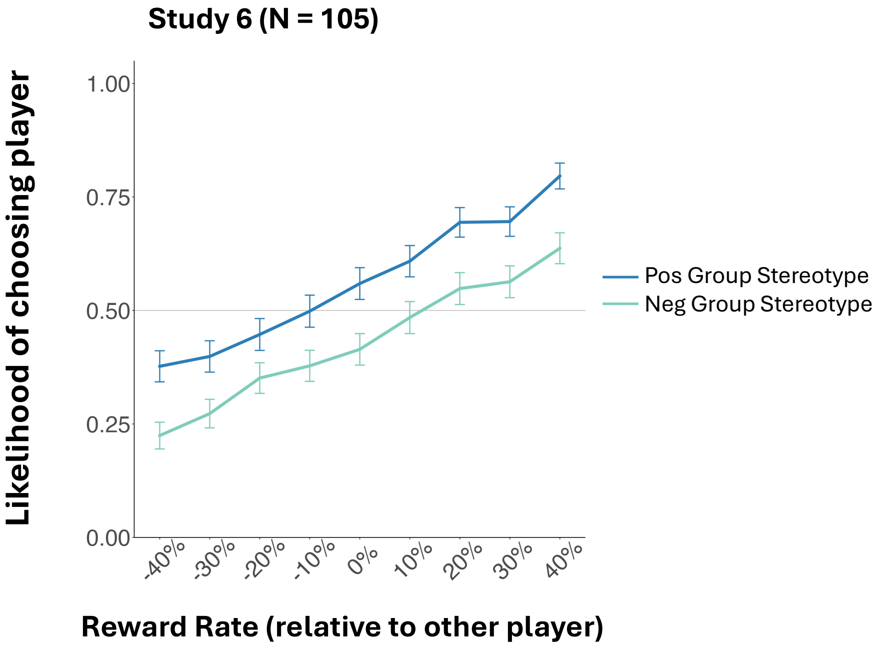

Explicit Rewards. Again, reported sharing rates were predicted by group membership, $B = 4.83$, $SE = 1.41$, $t = 3.42$, $p < .001$, and actual reward rates, $B = 46.05$, $SE = 4.46$, $t = 10.33$, $p < .001$. As in previous studies, the group effect remained significant when adjusting for explicit perception of rewards, $B = 0.48$, $SE = 0.03$, $t = 18.70$, $p < .001$.

Study 7

Overview. The purpose of Study 7 was to replicate the results obtained in Study 6, with one change: To ensure that participants understood that acting on the group stereotypes will reduce their earnings, participants had to pass an understanding quiz after completing the instructions and before starting the learning phase. Only if they correctly indicated that their explicit goal of maximizing rewards required focusing on individual players instead of stereotypes could they proceed to the task. Study 7 was preregistered at https://aspredicted.org/V8W_7ZC.

Method

Participants. Participants were 232 workers on the recruitment platform Connect who received $4.00 for their participation as well as a performance-based monetary bonus, ranging from $0 – $3.00, derived from points earned during the task. We excluded participants who failed to reach a learning criterion of 50% accuracy for 30%-70% player pairs during the test phase, as well as participants who did not finish the main task ($N = 78$). These exclusions resulted in a final sample size of $N = 154$ (87 men, 55 women, 1 nonconforming, 2 other, 9 unreported; $M_{age} = 39.12$ years, $SD_{age} = 11.24$ years).

Ethics approval was obtained from the human subjects institutional review board at the University of Amsterdam.

Procedure. Except for the novel understanding quiz, the procedure was equivalent to that of Study 6. The wording of the understanding quiz was as follows:

"Before you begin the main task, please answer this question about the task instructions:
To earn the most points in this task, I should base my choices on
-Descriptions of the player's group
-Feedback from individual players"

Results

Choice behavior. The quiz was correctly completed on the first attempt by 98 participants, by 53 participants on the second attempt, and by 3 participants on the third attempt. Our analytical approach followed that of previous studies: Multilevel regression predicting player choice showed significant effects of players' actual reward rate, *B* = 2.33, SE = 0.11, Wald *z* = 20.90, *p* < .001, as well as group membership, with participants preferring Group A members, *B* = 0.44, *SE* = 0.04, Wald *z* = 12.50, *p* < .001. The random slopes model produced a significant group effect as well, *B* = 0.55, SE = 0.22, Wald *z* = 2.51, *p* = .0012.

Explicit rewards. Reported rewards were predicted by group membership, *B* = 1.98, *SE* = 0.18, *t* = 11.20, *p* < .001, as well as reward rates, *B* = 41.02, *SE* = 0.55, *t* = 75.1, *p* < .001. Including explicit beliefs in the main analysis is consistent with previous results: Group membership predicts choices above and beyond subjective reports, *B* = 0.47, *SE* = 0.02, *t* = 26.00, *p* < .001.

Study 8

Overview. In Study 8, we tested whether a transmission of bias would occur between demonstrators from Study 7 and novel participants. In other words, we aimed to investigate whether prejudice would spread even when demonstrators explicitly tried to avoid the stereotype's influence. As in Study 5, novel participants observed Study 7 participants' behavior and subsequently made their own decisions. Study 8 was preregistered at https://aspredicted.org/H6M_SSZ.

Method

Participants. Participants were 300 workers on Connect who received $4.00 for their participation as well as a performance-based monetary bonus, ranging from $0 – $3.00, derived from points earned during the task. We excluded participants who failed to reach a learning criterion of 50% accuracy for 30%-70% player pairs during the test phase, as well as participants who did not finish the main task (*N* = 88). We also excluded participants who failed either of two attention measures, as in Study 5 (*N* = 88). These exclusions resulted in a final sample size of *N* = 154 (74 men, 72 women, 1 nonconforming, 2 other, 5 unreported; M_{age} = 35.72 years, SD_{age} = 10.53 years).

Ethics approval was obtained from the human subjects institutional review board at the University of Amsterdam.

Procedure. The procedure was equivalent to that of Study 5.

Results

Choice behavior. Again, multilevel regression predicting player choice showed significant effects of players' actual reward rate, *B* = 1.50, SE = 0.11, Wald *z* = 13.96, *p* < .001, as well as

group membership, $B = 0.19$, $SE = 0.03$, Wald $z = 5.67$, $p < .001$. The random slopes model did not produce a significant group effect, $B = 0.26$, SE = 0.27, Wald $z = 0.98$, $p = .32$. Furthermore, observer group bias was predicted by their respective demonstrator bias, $B = 0.15$, $SE = 0.05$, $t = 2.30$, $p = .003$. As in previous studies, adjusting for explicit rewards preserved the group effect, $B = 0.11$, SE = 0.16, Wald $z = 6.89$, $p < .001$.

Computational modeling

Our computational modeling analysis evaluated different hypotheses about the mechanisms underlying the group bias observed in the experiments. To this end, we adapted reinforcement learning (RL) and Bayesian learning models previously developed for understanding the influence on verbal instruction on learning (3).

Reinforcement learning. The basis for all reinforcement (RL) models was the standard Q-learning (or Rescorla-Wagner) learning rule:

$$Q_i^{t+1} = Q_i^t + \alpha(R^t - Q_i^t) \qquad [1]$$

where Q_i is the action value of selecting option i in trial t, R is the reinforcement [no reward = 0, reward = 1] received in trial t, and α ($0 \leq \alpha \leq 1$) is a learning rate parameter, which determines how much the difference between the received and the predicted reinforcement (the prediction error) affects subsequent value estimates (4).

In all RL models, the Q-values were transformed into decision probabilities using a standard Softmax function

$$P_i = \frac{e^{Q_i/\beta}}{\sum_{j=1}^{2} e^{Q_j/\beta}} \qquad [2]$$

where *β* (0.01 < *β* ≤ 100) is the temperature parameter that determines the sensitivity of choices to the difference in Q-values. Very low values of *β* results in selecting the action with higher Q-value with probability ~1, while high values of *β* result in explorative choices that are insensitive to the difference in Q-values. Together, equations 1-2 gives an unbiased standard learning model (model 1).

We considered two main mechanisms for group-based bias in RL. First, the semantic information provided in the manipulated group descriptions could result in different *priors*, or initial expectancies, about the value of selecting each group at the outset of the training phase. We implemented this by estimating a prior parameter, *P* (-100 ≤ *P* ≤ 100), which determined the initial Q value for the groups $Q_{Good}^{t=0} = prior, Q_{Bad}^{t=0} = -prior$). In models without this parameter, the initial Q-values were set to be equal ($Q_{Good}^{t=0} = Q_{Bad}^{t=0} = 0.5$). Non-zero values of the *P* parameter bias initial choices of the group with *P* > 0. We implemented a model with priors but no reward learning in the *bias prior* model (model 2).

In addition to models with such symmetric priors, we evaluated models with separate priors, in which initial expectancies for either group were allowed to vary independently, introducing an extra parameter but allowing for increased flexibility. Models with symmetric priors provided a better fit to the data and their output will be reported in later sections.

It should be noted that, if the model allows for reward learning, the influence of the *P* parameter decreases exponentially across training trials. In other words, experiential learning can rapidly counteract the initial expectancies. We evaluated this in the *bias prior RL* model (model 3).

Second, the *learning rate*, α, might differ between groups, so that participants update Q-values more (or less) rapidly from interacting with one group than the other (eq. 1). To evaluate biased updating, we either estimated α by group (2 α), or by both group and sign of the prediction error (4 α), based on classic social psychological theories that relate prejudice to differential attention to groups (i.e., ingroup favoritism, 49, and outgroup homogeneity, 50) and differential processing of positive and negative behaviors of ingroup vs. outgroup members (i.e., the ultimate attribution error, 51).

Bayesian learning. We also tested how Bayesian learning models accounted for the data. The main motivation for this approach is that Bayesian priors can have a stronger, more long-lasting effect on behavior than in the RL framework we describe above (where the prior is just the initial Q-value). We used standard Bayesian beta-binomial learning models (3), which explicitly estimate the probability of reward for selecting each group, given a beta distributed prior with hyperparameters α and β (both initialized to 1 for each stimulus *i*). The model learned by updating α and β (for each stimulus) by adding the running count of reward and no-reward feedback (separately for each stimulus *i*). Given a beta prior, this amounts to calculating the posterior distribution for each stimulus using Bayes rule:

$$\alpha_i^{t+1} = \alpha_i^{t} + pos \quad [3]$$

$$\beta_i{}^{t+1} = \beta_i{}^t + neg \quad [4]$$

where *pos* = 1 after reward feedback, and 0 after no-reward feedback, and vice-versa for *neg*. In addition, the running counts are decayed multiplicatively on each trial by a free parameter γ ($0 \leq \gamma \leq 1$), which allows the model to forget potentially outdated information (8). Choices were probabilistically taken (following a Softmax function, eq. 2) by comparing the modes of the posterior distributions:

$$mode_i = \frac{\alpha_i - 1}{\alpha_i + \beta_i - 2} \quad [5]$$

We evaluated two versions of this model. In the first version (model 9), the initial α parameter for Group A was estimated. In this model formulation, both the mode and the precision of the prior is affected by a. More evidence is required to counteract a precise prior. If a_{Good} is higher than a a_{Bad}, the model is biased to select group A. The second model (model 10) incorporated an additional parameter w ($1 \leq w \leq 100$), which modulated the feedback in a manner congruent with the semantic information (i.e., a confirmation bias). For stimuli from Group A, this gives

$$\alpha_i{}^{t+1} = \alpha_i{}^t + wpos \quad [6]$$

$$\beta_i{}^{t+1} = \beta_i{}^t + \frac{1}{w}neg \quad [7]$$

In other words, the model learns faster from positive outcomes and slower from negative outcomes. For stimuli from Group B, the effect of *w* was the inverse (i.e., faster learning from negative outcomes and slower learning from positive outcomes).

We exploratorily evaluated two Bayesian models for Studies 1-3, primarily to allow for a stronger influence of the prior. As they provided poor fit to the data, we did not evaluate these models in later studies.

Table S1. Overview of tested models.

Model #	Conceptual label	parameters	# parameters
**1**	*Unbiased learning*	*α,β*	2
**2**	*Stereotype-only*	*β, P*	2
**3**	*Stereotype-individuation*	*α,β,P*	3
**4**	*Group-learning*	α_{Good},α_{Bad},β	3
**5**	*Stereotype-learning*	α_{Good},α_{Bad}, P,β	4
**6**	*gain/loss group-learning*	α_{Good+},α_{Good-}, α_{Bad+}, α_{Bad-},β	5
**7**	*Stereotype-gain/loss group learning*	α_{Good+},α_{Good-}, α_{Bad+},α_{Bad-},P, β	6

Parameter estimation. Parameter estimation was conducted using the maximum-likelihood approach, which finds the set of parameters that maximize the probability of the participant´s trial-by-trial test phase choices given the model. Optimization was done by to minimizing the negative log-likelihood, -*L*, computed by:

$$-L = -\sum_{t=1}^{T} ln\,(P_{choice}(t)) \qquad [8]$$

where *T* denotes the total number of trials. Parameters were independently fitted to the test phase data for each participant using the Nelder-Mead optimization method. To avoid local minima in parameter fitting, optimization was initiated with 60 randomly selected start values. Model implementations and parameter fitting was done in *R* 3.5.1.

Model comparison. Model comparison was primarily based on the Akaike Information Criterion (AIC), a measure of goodness of fit of a model that penalizes complexity (9):

$$AIC = -2\ln(L) + 2k \qquad [9]$$

where *–ln(L)* is the negative log-likelihood and *k* is the number of model parameters. A smaller AIC hence indicates a better model fit.

Model comparison was based on the sum AIC across participants. For simplicity, we present the *ΔAIC*, which is the difference between model *i* and the best fitting model.

Table S2 shows the ΔAIC for each experiment separately, as well as the combined ΔAIC. Model 5, combining biased prior expectations and biased learning rates for each group, fit the data best in experiments 1-2, 4, and 6-7. Experiment 3 was best fit by model 7, which included separate learning rates for positive and negative prediction errors (equation 1) from group A and B. However, the difference between model 5 and 7 in Experiment 3 was relatively small. To formally test for the reliability of the apparent difference between experiments, we used a random-effects approach. Specifically, we used a linear mixed model with AIC as the dependent variable, and participant as random factor to test the interaction between experiment and model. This approach showed a main effect of model, $F(7, 3420) = 2.79$, $p = .004$, indicating that model 5 had significantly lower AIC than the other models. However, there interaction between model and experiment was not reliable, $F(24, 3420) = 0.83$, $p = .69$, indicating that model 5 provided the best fit across experiments. In addition, Bayesian model comparison indicated

that the posterior probability that all four experiments had the same model frequency was *P* = 0.96. Combining all experiments, we also find that the exceedance probability of model 5 was the most common among the candidate models was 1. Together, these results indicate that a combination of biased priors and biased learning rates best accounted for the influence of group descriptive information on instrumental learning across experiments.

Table S2. ΔAIC by model. The table shows the difference in AIC, summed across participants, for each model relative to the best fitting model (with ΔAIC = 0).

Model	1	2	3	4	5	6	7
**Experiment 1**	511	1140	213	175	0	252	228
**Experiment 2**	709	703	474	71	0	843	1141
**Experiment 3**	884	1536	196	199	70	114	0
**Experiment 4**	1397	2004	201	304	0	374	154
**Experiment 6**	743	1833	86	203	0	216	193
**Experiment 7**	1975	3392	640	692	0	677	306

Relation between model parameters and group-based bias. To understand in more detail how the parameters of Model 5 related to the stereotype bias observed in choice behavior, we regressed the estimated model parameters (excluding the Softmax temperature β) onto the degree of choice preference for positively-stereotyped group members in the test phase (proportion of *Group A* choices). All model parameters were rank transformed and standardized to improve linearity and interpretability. We conducted this analysis for Studies 1-3. We found that both the prior *P* (β = 0.084, SE = 0.012, t = 6.62, $p < .0001$) and the learning rate parameter for the negatively-stereotyped group ("Group B"; α_{bad}: β = -0.039, SE = 0.013, t = -3.7, p = .003) statistically predicted the degree of group preferences. In other words, a larger

initial value difference between the groups (the prior), together with a lower learning rate for Group B, was associated with a stronger bias. The learning rate for the positively-stereotyped group ("Group A") was not reliably related to bias (α_{good}: β = -0.003, SE = 0.011, t = -0.33, p = .74).

Supporting Information References

1. P. G. Devine, A. J. Elliot, Are racial stereotypes really fading? The Princeton trilogy revisited. Pers. Soc. Psychol. Bull. 21, 1139–1150 (1995).

2. A. P. Gregg, B. Seibt, M. R. Banaji, Easier done than undone: asymmetry in the malleability of implicit preferences. J. Pers. Soc. Psychol. 90, 1–20 (2006).

3. B. B. Doll, W. J. Jacobs, A. G. Sanfey, M. J. Frank, Instructional control of reinforcement learning: a behavioral and neurocomputational investigation. Brain Res. 1299, 74–94 (2009).

4. R. A. Rescorla, A. R. Wagner, “A Theory of Pavlovian Conditioning: Variations in the Effectiveness of Reinforcement and Nonreinforcement” in Classical Conditioning II: Current Research and Theory, A. H. Black, W. F. Prokasy, Eds. (Appleton- Century-Crofts, 1972), pp. 64–99.

5. M. B. Brewer, The psychology of prejudice: Ingroup love and outgroup hate? J. Soc. Issues 55, 429–444 (1999).

6. B. Park, M. Rothbart, Perception of out-group homogeneity and levels of social categorization: Memory for the subordinate attributes of in-group and out-group members. J. Pers. Soc. Psychol. 42, 1051–1068 (1982).

7. T. F. Pettigrew, The ultimate attribution error: Extending allport’s cognitive analysis of prejudice. Pers. Soc. Psychol. Bull. 5, 461–476 (1979).

8. Stephan, K. E., Penny, W. D., Daunizeau, J., Moran, R. J., & Friston, K. J. (2009). Bayesian model selection for group studies. Neuroimage, 46, 1004-1017.

9. N. D. Daw, “Trial-by-trial data analysis using computational models” in Decision Making, Affect, and Learning, (Oxford University Press, 2011), pp. 3–38
